# Supplementary material for: Combined activation of MAP kinase pathway and β-catenin signaling cause deep penetrating nevi
Source: Nat Commun. 2017 Sep 21;8:644. doi: 10.1038/s41467-017-00758-3 (PMC5608693; doi:10.1038/s41467-017-00758-3)
Supplement: Supplementary file 1 — Supplementary Information [file 41467_2017_758_MOESM1_ESM.pdf]

Description of Supplementary Files

File Name: Supplementary Information
Description: Supplementary Figures and Supplementary Tables

File Name: Supplementary Data 1
Description: Gene panel version 1 bait intervals.

File Name: Supplementary Data 2
Description: Gene panel version 1 gene list.

File Name: Supplementary Data 3
Description: Gene panel version 2 bait intervals.

File Name: Supplementary Data 4
Description: Gene panel version 2 gene list.

File Name: Supplementary Data 5
Description: Gene panel version 3 bait intervals.

File Name: Supplementary Data 6
Description: Gene panel version 3 gene list.

File Name: Peer Review File

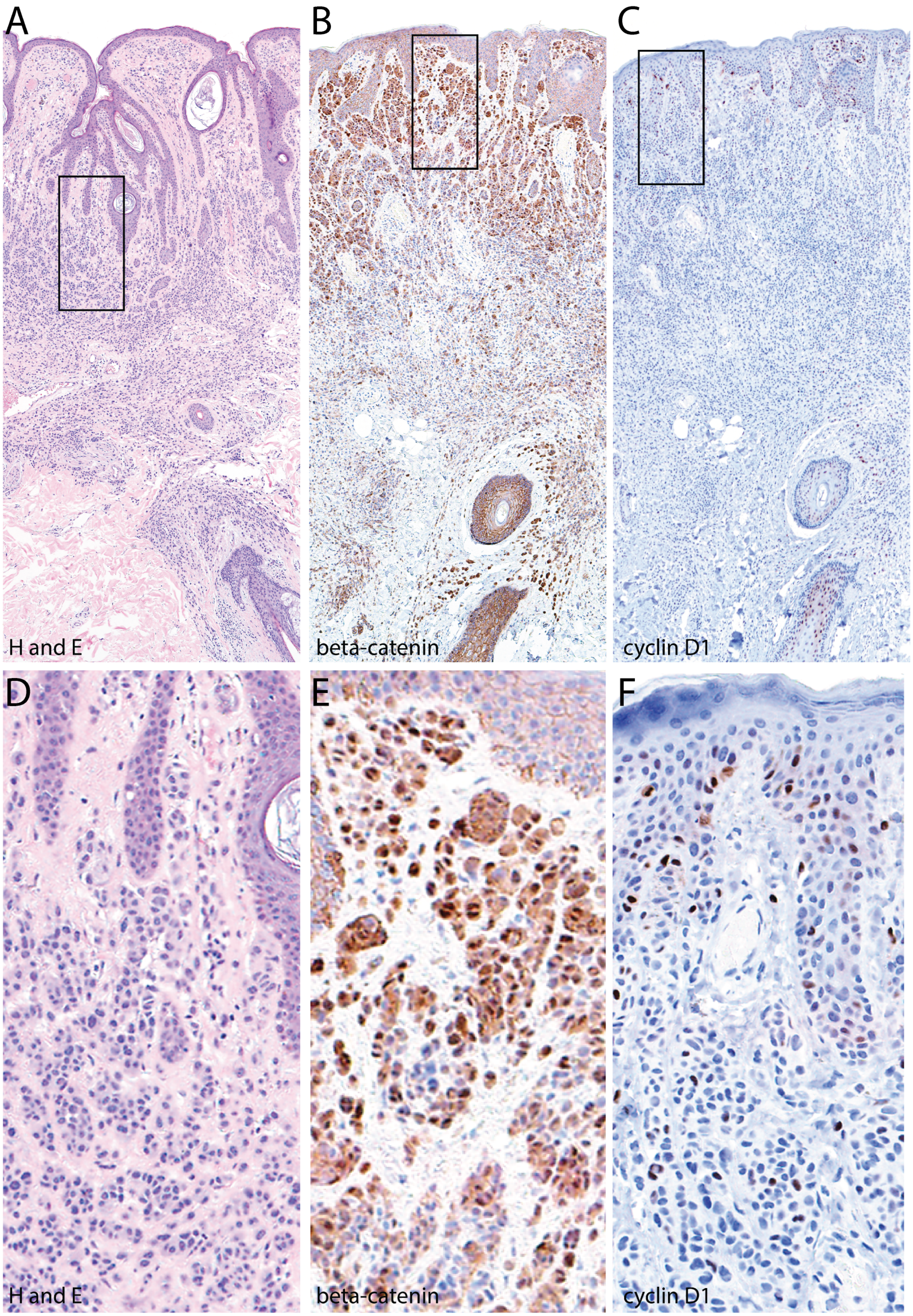

Supplementary Figure 1. In common acquired nevi, β-catenin and cyclin D1 expression is increased in melanocytes near epithelium. A. H and E of conventional nevus. **B.** β-catenin is present in melanocytes near the epidermis and follicular epithelium (arrows). **C.** Cyclin D1 is present in melanocytes in corresponding areas (arrows). **D-F.** High power views from region denoted by box in A-C.

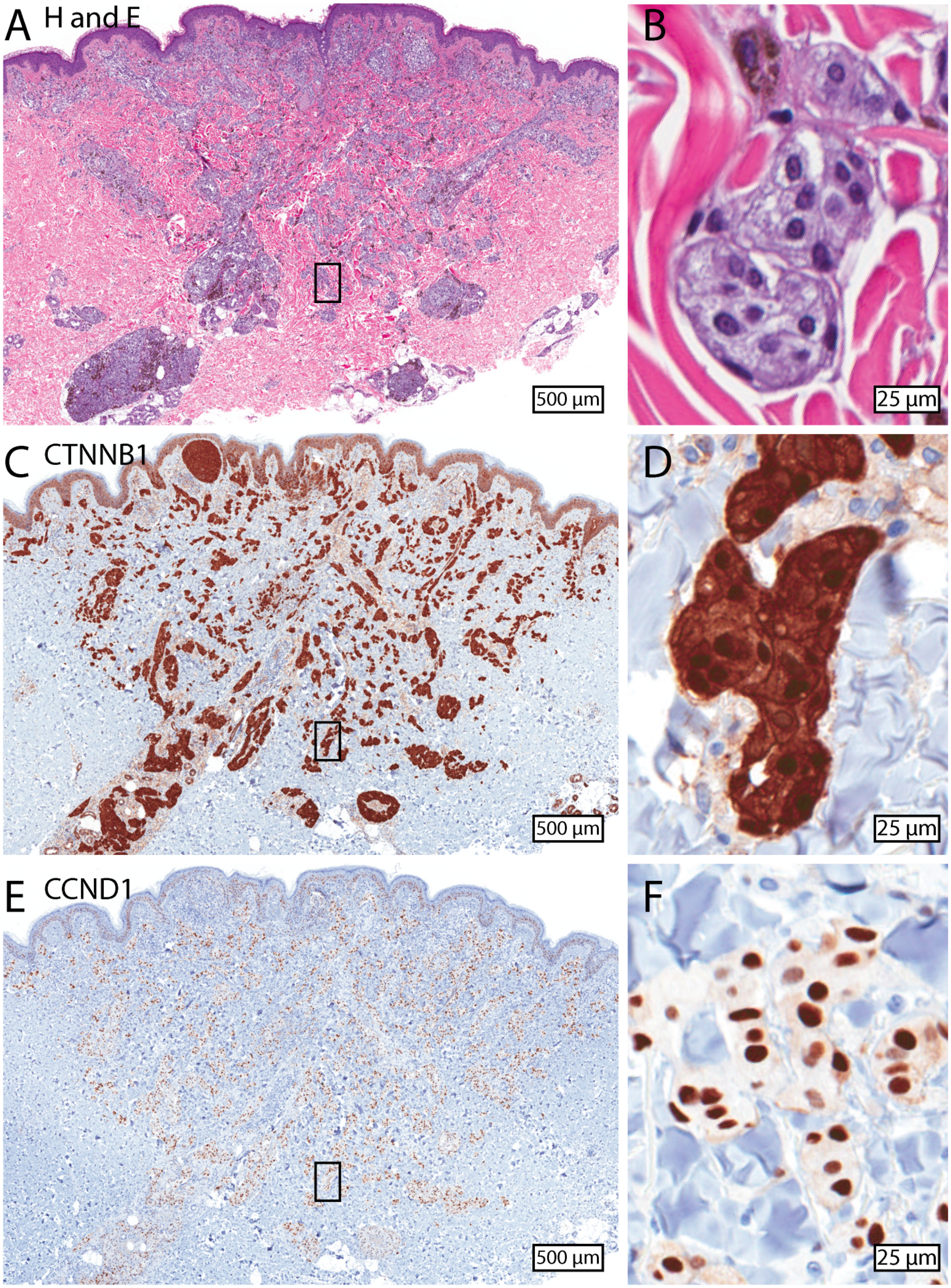

Supplementary Figure 2. Deep penetrating nevi (DPN) display uniform levels of β-catenin and cyclin D1. A. low power view of DPN with hematoxylin and eosin staining. **B.** high power view of DPN melanocytes within the deep dermis. The melanocytes have moderate amounts of vacuolated pigmented cytoplasm. **C.** low power view of DPN with immunohistochemical staining for β-catenin. Levels of β-catenin appear uniform within the tumor. **D.** high power view of DPN melanocytes in the deep dermis with immunohistochemical staining for β-catenin. There is cytoplasmic and nuclear staining. **E.**: low power view of DPN with immunohistochemical staining for cyclin D1. Levels of nuclear cyclin D1 appear uniform within the tumor. **F.** Nuclear staining for cyclin D1 is present in deep dermal melanocytes.

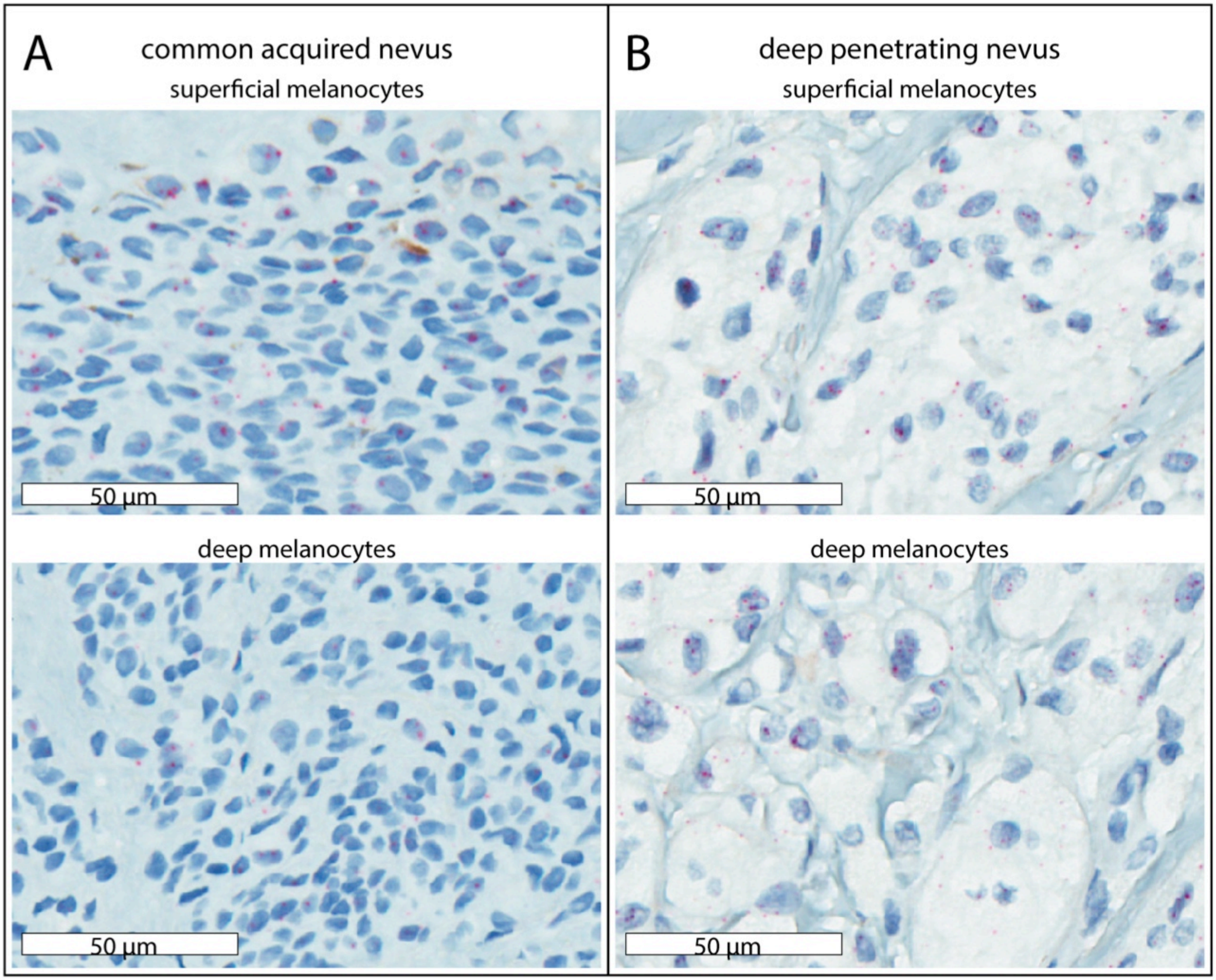

Supplementary Figure 3. *AXIN2* expression diminishes with depth within the dermis in common acquired nevi but not deep penetrating nevi. A. *AXIN2* expression is diminished in the deep melanocytes of a common nevus as compared to superficial melanocytes. **B.** *AXIN2* expression is similar in deep melanocytes of a DPN as compared to superficial melanocytes.

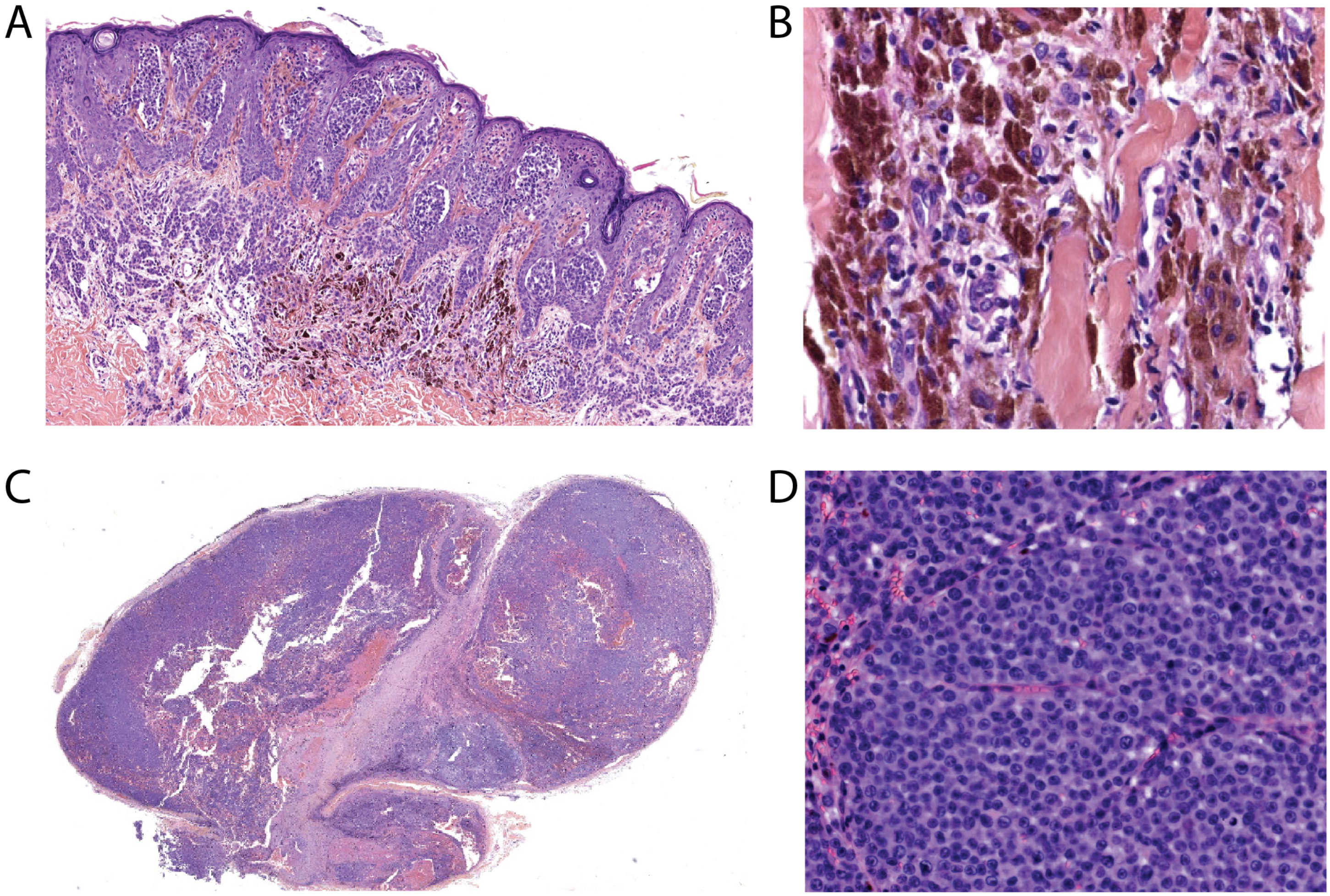

Supplementary Figure 4. Histopathology of DPN and matched metastasis (DPNMM102). A. Low power view of DPN on the chest of a 10 year old girl **B.** High power view of DPN. **C.** Low power view of metastasis to lymph node with effacement of lymph node architecture. **D.** High power view of metastatic deposit in lymph node.

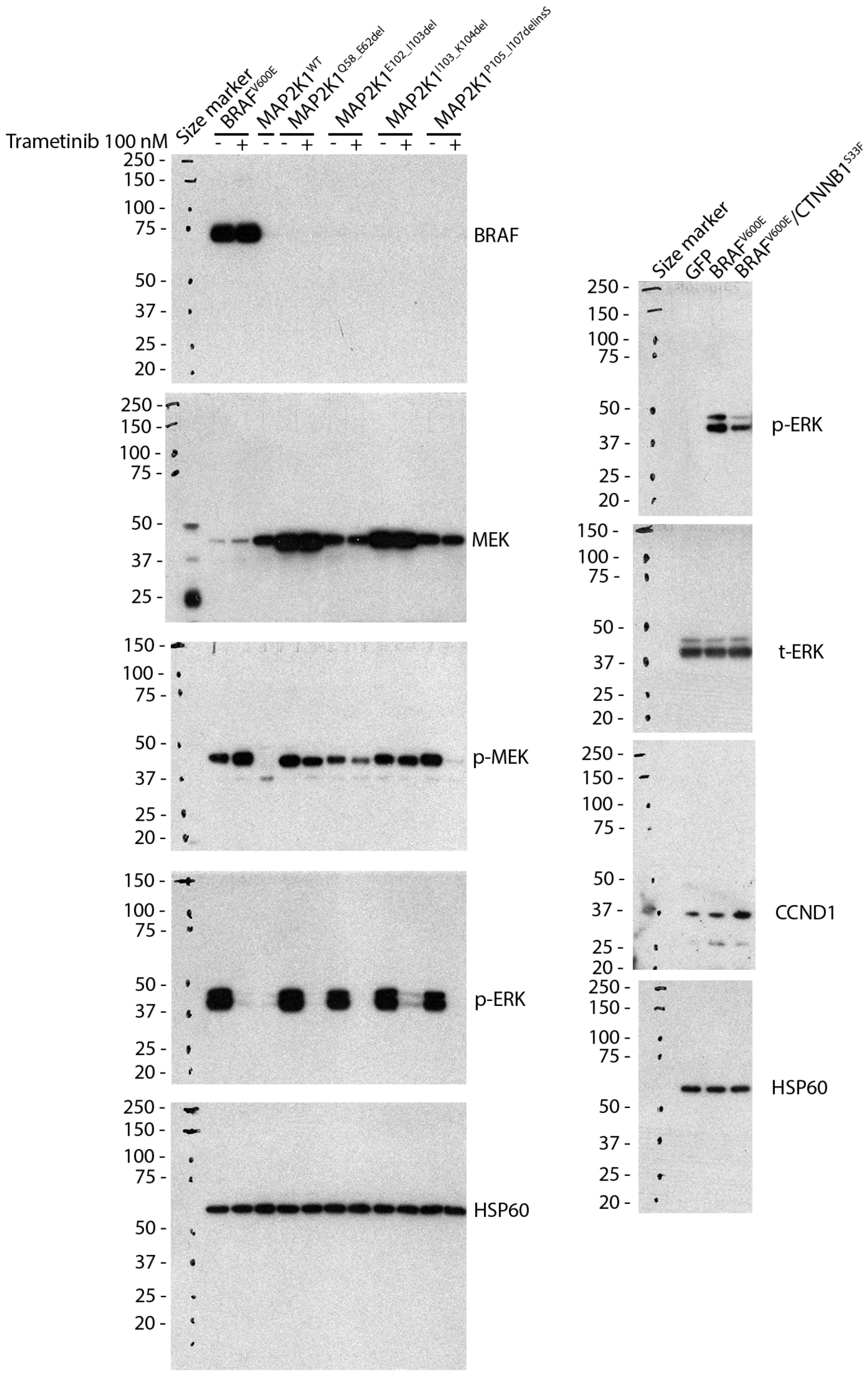

Supplementary Figure 5. Full western blots for Figures. 1D and 4.

Supplementary Table 1. Clinical Features and Pathogenic mutations in deep penetrating nevi (DPN), nevi with overlapping features of DPN and blue nevi and common nevi

Case	Nevus Type	Age	Sex	Site	BRAF	NRAS	HRAS	MAP2K1	CTNNB1	APC	GNAQ	IDH1
DPN3	DPN	41	M	back	AKAP9-BRAF	WT	WT	WT	D32N	WT	WT	WT
DPN10	DPN	29	F	shin	A598_T599insI	WT	WT	WT	S37F	WT	WT	WT
DPN60	DPN	25	F	nose	V600E	WT	WT	WT	D32H	WT	WT	WT
DPN16	DPN	15	M	forearm	V600E	WT	WT	WT	S37Y	WT	WT	WT
DPN57	DPN	24	F	back	V600E	WT	WT	WT	S45F	WT	WT	WT
DPN17	DPN	57	F	forearm	V600E	WT	WT	WT	T41A	WT	WT	WT
DPN7	DPN	32	M	leg	V600E	WT	WT	WT	T41I	WT	WT	WT
DPN50	DPN	11	F	cheek	V600E	WT	WT	WT	S37F	WT	WT	WT
DPN56	DPN	35	F	shoulder	K601E	WT	WT	WT	WT	S978fs and R1096*	WT	WT
DPN4	DPN	54	M	flank	WT	WT	WT	Q58_E62del	S33F	WT	WT	WT
DPN62	DPN	18	M	shoulder	WT	WT	WT	E102_I103del	S33F	WT	WT	WT
DPN18	DPN	20	F	shoulder	WT	WT	WT	I103_K104del	S33A	WT	WT	WT
DPN12	DPN	51	M	arm	WT	WT	WT	I103_K104del	S33F	WT	WT	WT
DPN8	DPN	29	F	chest	WT	WT	WT	P105_I107delinsT	D32N	WT	WT	WT
DPN49	DPN	27	M	neck	WT	WT	WT	P105_I107delinsS	S37F	WT	WT	WT
DPN53	DPN	61	F	leg	WT	WT	Q61R	WT	D32N	WT	WT	WT
DPN1	DPN	10	F	back	WT	WT	WT	WT	S33F	WT	WT	WT
DPN19	DPN	34	F	arm	WT	WT	WT	WT	WT	WT	Q209L	R132C
DPN14	Overlap	12	M	forearm	WT	WT	WT	WT	WT	WT	Q209L	WT
DPN6	Overlap	17	F	arm	WT	WT	WT	WT	WT	WT	Q209L	WT
DPN55	Overlap	31	M	thigh	WT	WT	WT	WT	WT	WT	Q209L	WT
DPN58	Overlap	31	F	forearm	WT	WT	WT	WT	WT	WT	Q209L	WT
DPN61	Overlap	16	F	hand	WT	WT	WT	WT	WT	WT	Q209L	WT
DPN51	Overlap	36	F	buttock	WT	WT	WT	WT	WT	WT	Q209Y	WT
DPN11	Overlap	6	F	Back	WT	WT	WT	WT	WT	WT	WT	WT
CN23	Common	51	M	face	V600E	WT	WT	WT	WT	WT	WT	WT
CN24	Common	25	F	neck	V600E	WT	WT	WT	WT	WT	WT	WT
CN25	Common	71	M	temple	V600E	WT	WT	WT	WT	WT	WT	WT
CN26	Common	52	F	back	V600E	WT	WT	WT	WT	WT	WT	WT
CN27	Common	32	F	back	V600E	WT	WT	WT	WT	WT	WT	WT
CN28	Common	29	F	chest	V600E	WT	WT	WT	WT	WT	WT	WT
CN30	Common	56	F	back	V600E	WT	WT	WT	WT	WT	WT	WT
CN31	Common	26	M	arm	WT	Q61R	WT	WT	WT	WT	WT	WT
CN32	Common	25	F	foot	WT	Q61R	WT	WT	WT	WT	WT	WT
CN33	Common	67	F	back	WT	Q61R	WT	WT	WT	WT	WT	WT

WT: wild-type

Supplementary Table 2. Axin2 RNASCOPE Semi-quantitative Scoring

Case	Axin2 in superficial melanocytes	Axin2 in deep melanocytes	Difference in Axin2 score (superficial-deep)	Distance of deep dermal melanocytes from epithelium	PPIB in epidermal keratinocytes (positive control)	DapB in epidermal keratinocytes (negative control)
CN25	1	0	1	0.5 mm	3	0
CN26	2	1	1	0.5 mm	3	0
CN28	2	1	1	0.5 mm	3	0
CN33	1	0	1	0.3 mm	2	0
DPN3	1	1	0	0.6 mm	2	0
DPN7	2	2	0	0.6 mm	2	0
DPN16	1	1	0	0.6 mm	2	0
DPN17	1	1	0	0.6 mm	1	0
DPN1	2	2	0	0.6 mm	2	0
DPN4	3	3	0	0.6 mm	3	0
DPN8	1	1	0	0.6 mm	2	0
DPN12	2	2	0	0.6 mm	2	0

Supplementary Table 3. Genotyping of biphenotypic tumors with deep penetrating nevus and common nevus components

Case	Genotype of Common component			Genotype of DPN component			Sequencing method
	BRAF (MAF)	MAP2K1	CTNNB1	BRAF (MAF)	MAP2K1 (MAF)	CTNNB1 (MAF)	
DPN35	V600E (0.41)	WT	WT	V600E (0.26)	WT	T41A (0.09)	Illumina paired end
DPN37	V600E (0.26)	WT	WT	V600E (0.28)	WT	S45F (0.12)	Illumina paired end
DPN5	NA	NA	NA	V600E (0.16)	WT	D32G (0.16)	Illumina paired end
DPN59	NA	NA	NA	V600E (0.36)	WT	S45P (0.35)	Illumina paired end
15000333	NA	NA	NA	V600E	WT	WT	Sanger
14017350	NA	NA	NA	V600E	NA	T41A	Sanger
14016570	NA	NA	NA	V600E	WT	T41I	Sanger
15001550	NA	NA	NA	WT	WT	S33F	Sanger
14003106	NA	NA	NA	WT	NA	D32G	Sanger
13012928	NA	NA	NA	WT	WT	T40A	Sanger
13012440	NA	NA	NA	WT	NA	S37F	Sanger

WT: wild-type, NA: not available, MAF: mutant allele frequency (only available for Illumina sequencing)

Supplementary Table 4. Clinical Features and Genetic Features of DPN metastases and DPN-like melanomas

Case	Specimen	Age	Sex	Primary Site	Clinical History	Pathogenic Mutations	Copy Number Alterations
DPNMM102	DPN metastasis (lymph node)	10	F	chest	Lymph node metastasis 12 years after diagnosis	NRAS Q61R<br>CTNNB1 S37A	gain of 1q, 6p, 8q<br>loss of 6q, 8p
DPN49Graz	DPN metastasis (lymph node)	58	M	back	Brain metastasis and death	MAP2K1 I103S and E203K<br>CTNNB1 S37F<br>TERT promoter c.-124 G>A<br>TP53 T86M	multiple aberrations with focal amplification on 11p and 20q
DPNMM20	DPN-like melanoma (primary)	71	M	upper back	NA	BRAF K601E<br>CTNNB1 G34R<br>APC c.729_2T>C<br>TERT c.−146 G>A<br>NF1 Q912*<br>CDKN2A R58* with LOH	multiple aberrations including gain of distal 6p and loss of 9
DPNMM22	DPN-like melanoma (primary)	68	M	back	NA	NRAS G13R<br>CTNNB1 S37P<br>KIT D816V	gain of 1q, 2q
RPA914	DPN-like melanoma (primary)	42	F	shoulder	NA	NRAS Q61K<br>APC p.E893* with LOH	gain of 19<br>loss of 5q, 9
DPNMM21	DPN-like melanoma (primary)	82	F	shoulder	NA	BRAF V600K<br>TET2 T212fs	none
DPN42	DPN-like melanoma (primary)	33	F	shin	NA	NRAS Q61K<br>TERT c.−146 G>A	gain of 1<br>loss of 9, 11, 14<br>deep deletion of CDKN2A
DPN46	DPN-like melanoma, reclassified as blue nevus like melanoma (primary)	69	M	arm	NA	GNAQ R183Q, BAP1 V188fs	loss of 3

Supplementary Table 5. Sequencing Quality Metrics

Sequencing ID	Mean Coverage (after deduplication)	Mean Insert Size	Platform Version
DPN3	244	172	1
DPN4	141	158	1
DPN6	71	169	1
DPN7	246	180	1
DPN8	324	182	1
DPN9	127	157	1
DPN10	279	177	1
DPN11	151	174	1
DPN12	124	169	1
DPN14	247	174	1
DPN16	273	184	1
DPN17	318	178	1
DPN18	322	185	1
DPN19	293	180	1
DPN49	506	247	2
DPN50	340	266	2
DPN51	487	247	2
DPN52	122	221	2
DPN53	549	189	2
DPN55	353	226	2
DPN56	226	194	2
DPN57	496	240	2
DPN58	422	236	2
DPN60	441	280	2
DPN61	167	165	2
DPN62	225	190	2
CN23	58	176	1
CN24	150	192	1
CN25	64	174	1
CN26	42	162	1
CN27	81	171	1
CN28	89	191	1
CN29	44	173	1
CN30	87	162	1

CN31	43	182	1
CN32	152	178	1
CN33	105	203	1
DPN35-common component	144	201	1
DPN35-DPN component	126	201	1
DPN37-common component	350	182	1
DPN37-DPN component	120	193	1
DPN5- DPN component	165	173	1
DPN59- DPN component	203	174	2
DPNMM21	323	204	1
DPNMM22	97	197	1
DPNMM20	282	175	1
DPN42	204	195	1
DPN46	409	196	1
DPNMM102	449	164	3
DPN49Graz	71	146	2
RPA914	426	195	3

Supplementary Table 6. RNA Scope 2.0 HD RED Semi-quantitative scoring criteria.

Score	Criteria
0	No staining or < 1 dot/10 cells
1	> 1 dot/10 cells to 3 dots/cell
2	4-9 dots/cell. None or very few dot clusters
3	10-15 dots/cell and < 10% of dots are in clusters
4	>15 dots/cell or > 10% of dots are in clusters
